# Medical interns in district health services: an evaluation of the new family medicine rotation in the Western Cape of South Africa

**DOI:** 10.1186/s12909-023-04605-6

**Published:** 2023-09-04

**Authors:** Lauren Hutton, Louis Stander Jenkins, Robert Mash, Klaus von Pressentin, Steve Reid, Jennie Morgan, Paul Kapp

**Affiliations:** 1 Department of Family and Emergency Medicine, University of Stellenbosch, Stellenbosch, South Africa; 2 Division of Family Medicine and Primary Care Department of Family and Emergency Medicine, University of Stellenbosch, Stellenbosch, South Africa; 3 Division of Family Medicine, Department of Family, Community and Emergency Care, University of Cape Town, Cape Town, South Africa; 4 University of Cape Town, Cape Town, South Africa; 5 Department of Family Medicine, University of Cape Town, Cape Town, South Africa; 6 Department of Family and Emergency Medicine, University of Stellenbosch, Stellenbosch, South Africa

**Keywords:** Internship, Family medicine, Training, Rotation

## Abstract

**Background:**

In 2021, South Africa introduced a 6-month internship rotation in family medicine, in the second year of a 2-year internship programme for newly qualified doctors. This was a major change from the previous 3-months training in family medicine, and expanded the training platform to smaller district hospitals and primary health care (PHC) facilities, many of which had never had interns. The medical disciplines in South Africa needed to know if this change in the internship programme was worthwhile and successful. The aim of this study was to assess the new family medicine rotation for medical interns at district health facilities in the Western Cape Province.

**Methods:**

Descriptive exploratory qualitative research included six intern programmes across the province. Purposeful sampling identified a heterogeneous group with maximum variation in experience. Overall, eight interns, four managers, four supervisors and four intern curators were included. Individual semi-structured interviews were audio-recorded and the transcripts were thematically analysed using the framework method and Atlas-ti software.

**Results:**

Four major themes emerged around the varied structure and organisational characteristics of the rotations, the orientation and arrival of interns, their learning during the rotation, and impact on health services. A programme theory was developed that defined the key inputs (i.e. infrastructure, communication, orientation, preparation, prior learning and guidelines), processes (i.e. model of the rotation, clinical training and supervision, clinical teaching), outputs (i.e. more independent decision making, approach to undifferentiated problems, approach to chronic care and continuity, development of procedural skills, approach to sequential coordination of care and referrals, working in a multidisciplinary team and inter-professional learning, integration of multiple competencies, as well as becoming more person and community orientated).

**Conclusions:**

The new rotation in family medicine was positively experienced by most interns, supervisors and managers. It should lead to improved quality of care, better preparation for obligatory community service, and an increased likelihood of considering a career in district level health services. This study will form part of an exploratory sequential mixed methods study that incorporates the key issues into a questionnaire for a descriptive survey of all interns in a subsequent study.

**Supplementary Information:**

The online version contains supplementary material available at 10.1186/s12909-023-04605-6.

## Background

Health systems need to develop a workforce that is accessible, competent and motivated to provide health services [1]. In South Africa, the new national human resources for health strategy has, as one of its goals, the desire to build “an enabled, productive, motivated and empowered health workforce”, particularly in the context of transforming the health service towards universal health coverage, which will include a functional district and primary health care (PHC) system [2].

Medical practitioners are a key part of the public sector PHC workforce and it is estimated that there will be a shortage of 2293 doctors in the country by 2025 [2]. In order to meet the gap in medical practitioners the government and universities have increased the number of medical students and their exposure to PHC in the undergraduate curriculum. Upon graduation, these newly qualified doctors enter an obligatory 2-year internship. A medical intern is a doctor in training, who has completed their undergraduate degree, and is working as a junior doctor through apprenticeship in a specified internship programme. The purpose of internship is for newly qualified doctors to practice under supervision in a training post so that they are safe and competent to practice independently [3].

The length and duration of internship differs across the globe, with a 2-year foundation programme in the United Kingdom [4], a 1-year rotation through the major specialities in New Zealand [5], and in the United States the first year of residency is the equivalent [6]. The most recent change to South Africa’s medical internship programme was the introduction of a 6-month rotation in family medicine, during the second year of training [7]. These six months are spent at district hospitals and PHC facilities, ideally under the supervision of a family physician.

In South Africa, the guidelines for medical internship have evolved over the past 70 years [8]. Up until 2004 internship was a 1-year requirement, with rotations through the major hospital-based specialities [9]. In 2004 the internship was extended to 2-years and for the first time a 3-month rotation in family medicine was required [10]. In 2021 the internship was again re-structured to require 6-months of family medicine in the second year [7]. This change necessitated the other hospital-based specialities each giving up one month of their rotations. Consequently, there is a need to evaluate whether the longer family medicine rotation has added value to the internship training. In our increasingly resource-constrained environment there is also a need to evaluate the costs and benefits of the entire internship programme [2].

Apart from a 2013 study in KwaZulu-Natal, evaluating whether internship training adequately prepared South African medical graduates for community service in district hospitals, and an older study in 2002, there has been little research on the training of interns for PHC in South Africa [10, 11]. A study in KwaZulu-Natal indicated that interns had variable supervision during their training, and less than 10% were positive about the conditions of training facilities [12]. A national survey indicated that supervision was the aspect of internship that interns would change the most, with 33% having performed a procedure for the first time without supervision and over half the interns having had a whole shift supervised by a medical officer with less than 3 years of training [8]. A more recent study among community service doctors (one year-post internship) in South Africa revealed that 28% of them never did an emergency caesarean section during their internship training [13]. The Academy of Science of South Africa (ASSAf) has recommended that universities should continue to play a role in internship, which should be renamed ‘Postgraduate Years 1 & 2’ [14]. This would signal a shift from seeing interns as the most junior doctors in service delivery to young professionals who need development and training in order to prepare them for the community service year and independent practice.

Although the Health Professions Council of South Africa (HPCSA) has defined standard outcomes for the family medicine rotation nationally, there is a significant variation in how internship programmes in the various health districts have organised their rotations. As interns complete this longer rotation for the first time it is important to evaluate whether the outcomes have been achieved, how interns and trainers experienced the rotation, what they actually learnt and whether the variety of models for organising the rotation make a difference. The aim of the study was to assess the 6-month family medicine rotation for medical interns at district health facilities in the Western Cape. Insights gained from this study will help shape the intern programme in the Western Cape with possible transferable lessons for other South African provinces.

## Methods

### Study design

This was an exploratory descriptive qualitative study utilising semi-structured interviews with interns, intern curators, trainers and managers. This study will form part of an exploratory sequential mixed methods study that incorporates the key issues into a questionnaire for a descriptive survey of all interns in a subsequent study (not reported here).

### Setting

As from 2021, the first year of internship is divided into 3-month rotations through internal medicine, surgery, paediatrics and obstetrics. All interns then spend half of the second year in a family medicine rotation and the remainder of the year rotating for 2-months each through orthopaedics, psychiatry and anaesthesia.

The specific aim and objectives for learning are set out by the HPCSA [3]. The aim of the rotation is to produce a generalist doctor, who at the end of internship will “have the knowledge and skills to function at a district hospital, with necessary access to supervision, support and referral systems; have the ability to work independently in ambulatory care, within the district health system; be able to manage all patients presenting for care at primary care facilities; and apply knowledge, skills and attitudes in the management of these patients, while collaborating with other staff and referral centres.” Medical managers, clinical managers and intern curators at accredited facilities, are responsible for ensuring that the HPCSA’s requirements are met.

In 2020 there were 660 interns, allocated across 12 training complexes in the Western Cape. Training complexes are based around central, regional or large district hospitals. The Western Cape has two central hospitals, five regional hospitals and four large district hospitals that organise internship programmes. Interns from these hospitals would then be placed in a variety of district hospitals or PHC facilities during the 6-months. Each site should have a designated supervisor and the whole rotation is coordinated by an intern curator.

Interns work within the public sector and are employed by the Department of Health in temporary training posts. After internship the doctors are obligated to complete one year of community service anywhere in the country, often in underserved areas. The public health sector serves most of the population who do not have health insurance.

### Study population

Two central hospital programmes (Groote Schuur and Tygerberg), two regional hospital programmes (George and Worcester) and two large district hospital programmes (Helderberg and Mitchells Plain) were purposively selected to give maximum variation. Four of these were in the metro health services and two in rural areas.

### Sample size

The planned interviewees included the intern curator, two interns, two supervisors and one facility manager from each of the six programmes, giving a sample size of 36 respondents. The final sample size was determined by saturation of data from each of these groups. Data saturation was achieved if no new themes were identified in the last two interviews. If necessary additional interviews were conducted to ensure in-depth exploration of the topics.

### Sampling

Purposeful sampling was used to identify the participants with the intention to interview a heterogeneous group with maximum variation in experience. Half of the intern participants were selected from the first rotation of 2021 and half from the second rotation. Where possible the two interns selected from each programme were exposed to different district hospitals or PHC facilities. An equal number of male and female interns were selected and if there was an intern representative for the group, they were selected. Supervisors were selected from both PHC and district hospital sites in each programme. One manager from each programme was selected to ensure three managers from PHC facilities and three from district hospital facilities as well as a mix of urban and rural areas. Potential participants selected in this way were then invited to participate voluntarily.

### Data collection

Two interview guides were used, one for the semi-structured interviews with the interns, and one for the curators, managers and supervisors (see Appendix A and B). The interview guides explored the following topics:

- What was the educational model and structure of the programme.
- What did they learn and how well did this prepare them for community service.
- What was the quality of supervision.
- What was the impact on health services and service delivery.

Interviews were held towards the end of the second rotation in October and November 2021. Interviews were conducted by members of the research team as well as by a trained research assistant experienced in qualitative interviewing. Interviewers did not interview interns or colleagues from the same programme that they were involved with. Interviews could be held face-to-face or virtually using Zoom or Microsoft Teams©. Interviews were conducted in English, the official language for communication in the health services, and were audio-taped. Interviews were conducted at times and places that were mutually convenient (outside of working hours) and were approximately 30 to 60 min in length.

### Data analysis

Recordings were transcribed verbatim and checked for any errors. The transcripts were thematically analysed using the framework method and with the help of Atlas-ti version 8© [15]. Transcripts were divided between four of the researchers for analysis (LH, RM, LJ, SR). The following steps were followed:

#### Step 1

Researchers familiarised themselves with their transcripts and identified issues to include in the analysis.

#### Step 2

Researchers met and reached consensus on a coding index. Codes were defined and organised into categories.

#### Step 3

Researchers independently coded their transcripts in Atlas-ti using the same coding index. If necessary new codes were added. All coded transcripts were then merged in Atlas-ti from the different researchers.

#### Step 4

Code families were created to bring together codes on similar topics as per the categories from the coding index. A report on all the data was generated for each code family. Reports were distributed between the researchers.

#### Step 5

Researchers independently interpreted their reports and wrote up their section of the findings. The findings were then integrated by RM and shared with all the researchers who reviewed and agreed on the final interpretation. Each researcher added quotations from the data to support the interpretation.

### Trustworthiness

The researchers were all family physicians who were interested to evaluate the rotation. RM was an academic family physician at Stellenbosch University who used to be the family medicine intern curator at Tygerberg Hospital, but was no longer directly involved. He was an established researcher with experience of qualitative research. LH was a newly qualified family physician in Knysna District Hospital. This was a rural setting, where she coordinated the interns at the district hospital. LJ was a family physician at George Regional Hospital and the family medicine intern curator. He has substantial experience in qualitative research. SR was an academic family physician at University of Cape Town, he was not directly involved in the internship programme, and was an established researcher with experience of qualitative research.

Apart from peer reviewing each other’s interpretation of the data, findings were also presented to family physicians from both the rural and urban platforms for their feedback and comment. The other researchers, KvP, JM and PK, not directly involved in the analysis, also peer reviewed the interpretation.

## Results

### Characteristics of respondents

The characteristics of the respondents are outlined in Tables 1 and 2. Overall there were 21 interviews, eight with interns, four with supervisors, five with curators and four with facility managers.

**Table 1:** Interns characteristics

	Gender	Semester	Base Hospital
1	Female	1st	George
2	Female	1^st^	Tygerberg
3	Male	1^st^	Worcester
4	Female	2^nd^	Mitchells Plain
5	Female	2^nd^	George
6	Male	2^nd^	Groote Schuur
7	Female	2^nd^	Helderberg
8	Male	2^nd^	Mitchells Plain

**Table 2:** Supervisors characteristics

	Gender	Role	Relationship to interns	Hospital
1	Male	Family Physician/Clinical Manager	Manager	George
2	Female	Medical Officer	Curator	George
3	Male	Family Physician/Clinical Manager	Manager	Helderberg
4	Female	Medical Officer	Curator	Mitchells Plain
5	Female	Family Physician	Curator	Groote Schuur
6	Male	Clinical Manager	Manager	George
7	Male	Family Physician	Curator	Tygerberg
8	Female	Manager	Manager	Worcester
9	Female	Paediatrician	Curator	Worcester
10	Female	Family Physician	Supervisor	Helderberg
11	Male	Family Physician	Supervisor	Mitchells Plain
12	Female	Family Physician	Supervisor	Tygerberg
13	Female	Emergency Medicine Physician	Supervisor	Worcester

### Overview of themes

The following major themes were identified:

- Structure and organisation of the training rotations.
- Orientation and arrival of the interns.
- Learning during the rotation.
- Impact on health services.

### Structure and organisation of training rotations

Table 3 shows the varied structure of the rotations at different sites. Most rotations exposed interns for two months to PHC facilities, where they saw ambulatory undifferentiated patients and patients attending for chronic diseases. Tygerberg interns had the largest commitment to PHC and interns performed overtime at the 24-hour community health centres. George interns were allocated to the district hospitals and some then rotated interns for a 2-month period to PHC, while others released them 1–2 days per week to PHC.

**Table 3:** Structure of intern rotations

Training site	Primary care facility (months)	District hospital (months)	Regional / central hospital (months)	Specialised hospital* (months)
Helderberg Large District Hospital	2	4	0	0
Mitchells Plain Large District Hospital	3	1	0	2
Groote Schuur Central Hospital	2	3	1	0
Tygerberg Central Hospital	4	2	0	0
Worcester Regional Hospital	2	2	2	0
George Regional Hospital	2	4	0	0

* This was a specialized TB hospital

Interns spent between 1 and 4 months at the district hospital where they worked in outpatients, the general wards, labour ward, operating theatre, and the emergency centre (EC). In the Worcester training site, the district chose not to place interns at the district hospitals as they were concerned about the additional supervision that would be needed. Interns were subsequently sent to a district hospital in a neighbouring district.

Three of the sites retained interns for 1–2 months in the central or regional hospital, where they were allocated to the EC. The rationale for this was that they would see the undifferentiated patients who were similar to patients seen in PHC. One site allocated interns to a specialized TB hospital for 2 months, which drew criticism from the interns as it was not perceived as relevant to the HPCSA outcomes. Overall, some interns felt there was too much exposure to the EC environment versus PHC:

“So the CHC part was mostly, we spent a lot of time in the trauma unit and then we also spent some time seeing patients in the out-patient department… I find that we had too much experience on emergencies and not enough focus on primary health” [D12: Intern 2, Helderberg programme].

Interns fulfilled their overtime obligations in the EC of the hospital or 24-hour community health centre. There were typically five to six calls a month. In some locations, interns worked through the night until 12h00 the next day (28 h on duty), while in other sites they worked until 23h00 and then went home. In all locations, they contributed an average of 60 h overtime per month.

Most interns and managers agreed that it was better to engage with the rotation in the second year when interns had completed the other hospital-based specialities. They were then better prepared to deal with the generalist environment, apply what they had learnt and manage undifferentiated patients. Some even thought it was ideal for this to be the last rotation in the two-year programme:

“…they go in their second year, so first I need to rotate through the basic speciality at Worcester Hospital, which I think is a good thing, because it prepares them for when they do the family meds rotation, you do get your more general under, undiagnosed, undefined patients that I don’t think a junior first year intern will have the clinical reasoning or ability to make clinical decisions [on] in the first year **[**D19: Supervisor, Worcester programme].

Managers chose the specific sites for training, with regard to HPCSA accreditation, the availability of sufficient senior supervision, the likelihood of a good learning environment, enough space, and the presence of other learners who might compete for supervision. It was particularly important to choose training sites with stable services and senior supervision. District hospitals with a history of staffing and management instability were excluded from intern rotations. Having a family physician at the site to supervise and coordinate training was seen as ideal:

“I think Hermanus is the only stable, level one district hospital, where services [have] senior presence in the Hermanas hospital, it’s well organised, well planned. And I think that’s the only reason. Ceres has since I have been here, I am here nine years now, Ceres has forever been a problem and there is no stability” [D17: Manager, Worcester programme].

Several of the hospital-based specialities felt that interns should not take leave during these rotations (which were now three months long) and thus implied that they should use their leave during the family medicine and PHC rotation:

“…biggest complaint is just make sure that the interns don’t get leave in that shorter rotation now because then it’s even worse. So there’s a little bit of planning to make sure that at least they will be able to use time to the max benefit, in the short time that they’ve been allocated.” [D17: Manager Worcester, programme].

### Orientation and arrival of interns

Interns initially felt anxious about being away from their main hospital, but this became less with subsequent interns rotating out. To alleviate intern uncertainty and anxiety, intern curators met with district hospital managers and family physicians and planned the new rotations in detail, attempting to accommodate intern and district hospital expectations. Finding new accommodation and the cost of moving there and back again was an issue in some sites:

“…you had to go and then you had to come back, find a new accommodation, move all your stuff again…It shouldn’t be costing us a whole lot of extra money.” [D5: Intern 1, George programme].

Intern curators were responsible for the whole rotation, while supervisors were based at the various facilities. Curator were usually located in the main hospital and some distance from the supervisors. Monthly meetings between the curator and supervisors helped to maintain open communication and early identification of issues with interns. The interns’ relationship with the curator was variable with complaints of poor communication “*the communication was not desirable*” and availability. There was some confusion in terms of the roles of the curator versus the local supervisors. The curator-supervisor-intern teamwork appeared stronger in the rural sites with more support for interns:

“And yes, there was no, there was basically no communication … we didn’t really have any input …” [D6: Intern 1, Tygerberg programme].

“My intern curator at Worcester Hospital is excellent…made us feel like we you know that we had rights and that we could stand up for them and she would stand up for us…” [D7: Intern 1, Worcester programme].

The usefulness of orientation on arrival for the rotation as a whole differed between sites, with some having a comprehensive and structured induction, while others appeared brief, unstructured and unsatisfactory. This left interns uncertain about what was expected. Orientation also varied between facilities and even sub-sections of facilities within the same rotation:

“But they actually did a whole session with us the day before we started working. And then the day of that week, actually every day we got like a session, orientating us in different parts of the hospital.” [Intern 1, George programme].

“…in terms of the orientation. Yes, because like we kind of started it, we were kind of like thrown into it you know, we didn’t really to know what to expect or what was expected of us**…** I think a better orientation would have helped because we were sort of finding out things along the way…” [D6: Intern 1, Tygerberg programme].

### Learning during the rotation

#### Development of autonomy and clinical decision making

During this rotation, interns were given more responsibility for patient care and had to make their own clinical decisions. In the tertiary setting, as part of a larger team, they were often more responsible for drawing blood, administration and organization of care, where decisions were made by others. In this rotation, they learnt to be more independent, and this professional development was seen as better preparing them for community service. In PHC, the responsibility to sort out the problem is more on the single attending doctor and not a shared responsibility as in a hospital ward; in addition, the doctor cannot discharge the patient to someone else:

“We give them I think the freedom that’s a difference for them here. From what I hear from them, … is that there’s nobody looking over their shoulder you know trying to like guide them with every little detail.”[D14: Manager, George programme].

“I can tell you the second year, interns across the board, the first year, they still need to be babied and but by the second year there is just this, as a self-confidence. And yes, I think they kind of find themselves as doctors in the second year.” [D 17: Manager, Worcester programme].

#### Learning new clinical skills

They were exposed to chronic medical conditions and ongoing care and appreciated the value of continuity of care. In particular, they learnt how to provide ongoing care for patients with HIV, TB, diabetes and hypertension. In the one programme the interns felt there was too much emphasis on TB hospital type care:

“Brooklyn Chest, but it still was very much in-patient physician internal medicine work, it wasn’t by any means family medicine.” [D9: Intern 2, Mitchells Plain programme].

They were also exposed to acute medical conditions, not seen at the main hospital, for example, dermatological conditions. They were able to practice a range of procedural skills including suturing, excision of “lumps and bumps”, incision and drainage of abscesses, closed reduction of fractures, circumcisions, and insertion of chest drains. Some of these procedures were more easily practiced in the district health services setting. They were also able to gain further experience of procedures that had been learnt in the main hospital such as caesarean sections, general anaesthesia and spinal anaesthesia. They were exposed to a broad range of emergencies when on-call, including resuscitations, acute coronary syndromes and multiple trauma. When on-call in the district hospital interns had to cover the whole hospital from the labour ward to the EC. There was little mention of interns being involved in audits and feedback or other clinical governance activities.

#### The value of exposure to district health services and primary health care

Seeing undifferentiated patients meant interns had to consider a broad differential diagnosis and draw on knowledge across the disciplines from year one of the internship programme. They had to consider the reason for this encounter, which could be because of severe symptoms, concerns about symptoms, psychosocial problems or the need for disease prevention. Managing these patients helped to integrate knowledge around a particular presentation and develop an approach to common problems. Consecutive patients also required incorporating knowledge from different disciplines.

Dealing with undifferentiated patients required the ability to cope with uncertainty and complexity in the clinical process. Interns learnt to prioritise what to focus on and that not everything was urgent. In addition, they had to handle patients who were diagnosed for the first time or at an earlier stage in the natural history and evolution of their condition. Learning how to deal with these patients prepared them better for community service. It was also hoped that exposure during this rotation would change negative attitudes to PHC that were often nurtured in hospital environment:

“So I can say that overall a positive experience. I think it’s useful that we got to spend more time doing Family Medicine, seeing as that’s in a way kind of what we’re supposed to be trained for” [D7: Intern 1, Worcester programme].

Working in PHC enabled more awareness of patients’ community and family contexts, including exposure to community-based services such as retirement homes and palliative care facilities. Some interns enjoyed being immersed more in the realities and struggles of particular families and communities, with the opportunity for relational continuity and building relationships with patients. Communication skills became more important and some felt more exposure to the family physician would help improve these skills. They also had the opportunity to work with community health workers and do home visits.

#### Clinical training and supervision

Overall, the quality of supervision was perceived as good. Interns received clinical support and supervision from medical officers, family physicians and nurses; and all were seen as role models in different settings. Particularly in the rural areas, they became part of the local team very early:

“…we were a group of about 18 clinical doctors, plus minus, 18 to 20 with all of them included, interns included. So, it’s a small team and we … our group is you know we have a very supportive group…” [D14: Manager, George programme].

The majority of the clinical learning was from the medical officers at the district hospitals. While the supervision given to interns was strong and very present, there was a concern that the medical officers themselves may not be getting the teaching and supervision that they also needed; especially in order to be able to teach and guide the junior doctors in training:

“Or you know, and this is what the MOs said, like they’re there and we’re making them see patients and we, they present to us, and we help them, but we’re not sure what we should be teaching them you know.” [Manager, George programme].

Family physicians were integral parts of the supervision as they were available, approachable and had a wealth of knowledge in order to teach interns about family medicine. The quality of broader mentorship was low in most district hospitals.

Nurses introduced an interesting inter-professional learning opportunity in PHC and the district hospital. In some settings, visiting specialists and allied health professionals also provided learning opportunities. In the district health services, interns were exposed to health professionals that they had not previously engaged:

“…where they never had the opportunity to do so other than on ward rounds. Here it was a daily experience for them to walk the patient to the physiotherapist, to see what the social worker does …” [D15: Curator Tygerberg].

Supervision and support was available, but might be less intense and less immediate than in the main hospital. Supervision was stronger in the district hospital than in PHC. There were some reports of no supervision at PHC and the need for interns to manage complex clinical cases alone. There was mostly always advice available over the phone with local supervisors or referral centres.

Interns had to be more proactive in seeking the support and supervision they needed, as there were fewer health practitioners in the district clinical environment. It was important to balance the risks involved in giving interns more autonomy, with the need for supervision. Sites varied in their approach to this with some appearing overprotective and others over-exposing interns to risk, particularly in PHC.

Supervision was less present after hours and more likely to be provided by community service doctors and junior medical officers, but senior supervision was always available off-site. This seemed standard at all sites and likely reflected the way overtime was structured at the district level.

Family physicians were seen as role models for patient care in the district health services. They also brought registrars into the system who were important supervisors for interns and were more learning focused. Family physicians often took responsibility for organising and coordinating the whole rotation at the site as well as creating a learning environment across the whole facility. One setting in particular had explicitly developed a culture of learning, mentoring and critical thinking that cascaded through the team from the family physician to the intern.

#### Clinical teaching

The learning environment was seen as less structured than in the main hospital. The focus was more on clinical training with a patient, than academic meetings. It may be more difficult to monitor learning outcomes and topics covered in this less structured environment, but there were “*so many opportunities to learn*”. As interns rotated between different settings it was also difficult to ensure that each group had the same opportunities for clinical teaching:

“The academic discussions for instance are okay, a nice to have, but I think the more informal in-work training is like more effective and it maybe makes more of a difference and… persons learn more if there’s a hands on sort of approach.” [Manager, George programme].

Interns joined in with the routine educational activities such as academic ward rounds, continuing professional development (CPD) activities, morning huddles, and morbidity and mortality meetings. Some sites had dedicated weekly educational meetings with the interns, but not all programmes. In one setting, the family physician combined educational initiatives for all junior doctors. Some programmes established virtual educational activities across the whole platform that all interns could attend.

#### Navigating the health system and referral pathways

Interns took time to adjust to the district environment as the resources (e.g., medications, investigations, staffing levels) and model of care were different. In this setting, they learnt who needed to be referred and why. They also experienced how difficult it could be to refer a patient or make an appointment, and how the district hospital and PHC platform were intimately linked. The importance of coordinating care between levels became visible. Their exposure to the constant high workload and pressure contributed towards the development of coping skills and resilience:

“The guys were very excited about seeing primary care in the clinics, seeing what’s like outside, also a level 1 hospital. Experiencing the lack of… where they don’t have everything, we have in level 2. And it gives them a lot of empathy and also sympathy for the grassroots and level 1 referring to us.” [Curator, Worcester programme].

#### Use of the HPCSA logbook

Supervisors appreciated that the HPCSA logbook guided them on what learning opportunities were needed and helped them to identify interns in difficulty. At some sites, they regularly checked progress against the logbook. Interns agreed that the logbook guided them in what was expected, although some did not see its educational value. The alignment of HPCSA expectations and learning opportunities differed between programmes. In some programmes, the interns reported that they easily achieved most of the requirements, whereas in others they struggled. Some interns saw the logbook as a necessary evil that had to be completed, often with a lot of pressure at the end of the rotation.

#### Impact of COVID-19 on learning

This first rotation occurred during the COVID-19 pandemic and interns were diverted from the planned learning opportunities to help with testing, field hospitals, COVID-19 wards and vaccinations. In several programmes, the exposure to PHC was sacrificed to enable this. At the same time, patients with non-COVID-19 conditions were less likely to be seen in the EC and many services were cut back, such as surgery, which also reduced learning opportunities. There was also less exposure to continuity of care and follow-up of patients. In addition, routine CPD activities were cut back or stopped. Educational meetings for interns continued virtually in some programmes, although some interns felt that there was more participation in face-to-face meetings:

“And I think the, the main obstacle and the thing we probably didn’t do well was to get them into primary health care a lot and I think one doesn’t want to use Covid as an excuse every time, but we, for a long time we had to use the interns to run the swabbing station where we previously had nursing staff doing it and they all got pulled away to start doing, in the second half of the year, to start doing vaccinations.” [Clinical Manager, George programme].

### Impact on health services

Generally, the impact on health services was reported as being overwhelmingly positive, due to the extra pairs of hands to do the work. The impact was seen in a number of ways, and examples were given such as longer and better quality consultations with patients, more reliable assistance in the operating theatre, and more frequent visits to outlying clinics than before. There were frequent statements such as *“just basically having an extra pair of hands has made a difference”.* Some felt that this contributed to a better quality of care by allowing for more time with each patient. From a management perspective, the additional interns at district level gave the medical teams more options for allocating staff to the various roles. The net addition of medical staff was complemented by the clear inclusion of the interns as fully-fledged members of the team. The interns unanimously felt welcomed and supported and were able to integrate easily into their medical teams, as well as with the nursing and therapy staff.

There were some downsides and a few conditional responses, however. While one stated *“we are there, we are working hard, making the numbers less”*, some interns felt that they could *“ease the load, but we shouldn’t carry the load”*, indicating that they were used to fill gaps and felt unsupported at times. The clinical opinions of a few interns working at PHC level were not trusted by their hospital-based colleagues. Sometimes there was not enough physical space such as consulting rooms for the interns to see their own patients, and others were *“relegated to working with the nurses”* when they were additional to the requirements:

“…maybe send them to the bigger clinics which is the nicer ones like the [community day centres] CDC’s and it’s purely because the smaller clinics don’t always have the space for two doctors.” [D2: Manager, George programme].

The extra personnel also created a greater management burden, as one manager put it: *“extra bodies can be a lot of extra work”.* Some had to be specifically managed in terms of the organizational culture and expectations, for example seeking permission before leaving work in the afternoon. One particular group of interns required tight management in terms of professional behaviour such as coming late or taking extended lunch breaks, but this appeared to be a minority.

Attitudes towards family medicine as a specialty were mixed, with some enjoying it while others were negative. Clinical managers felt that it was an advantage for interns to learn how the system worked and appreciate the lack of resources at PHC level, which changed their perspectives regarding referrals when they returned to larger hospitals. There was no evidence that interns drove up costs in the district health services through unnecessary prescribing or laboratory tests, as reported by one manager who monitored expenditure closely. Although concerns were expressed in the regional hospital EC that they ordered too many investigations. Likewise, managers stated that there was no increase in patient complaints due to the interns. There was a clear benefit after hours when there was an extra doctor on call, especially in district hospitals where there had previously been only one medical officer after hours.

In terms of professionalism, setting expectations clearly at the beginning was important, as well as having good role models and inducting the interns into a group culture that took values seriously, and the need to “*hold each other accountable to be professional”*. Ultimately, the hope was that the interns knew their limitations in terms of when to refer or ask for help.

## Discussion

### Summary of key findings

**Fig. 1:**
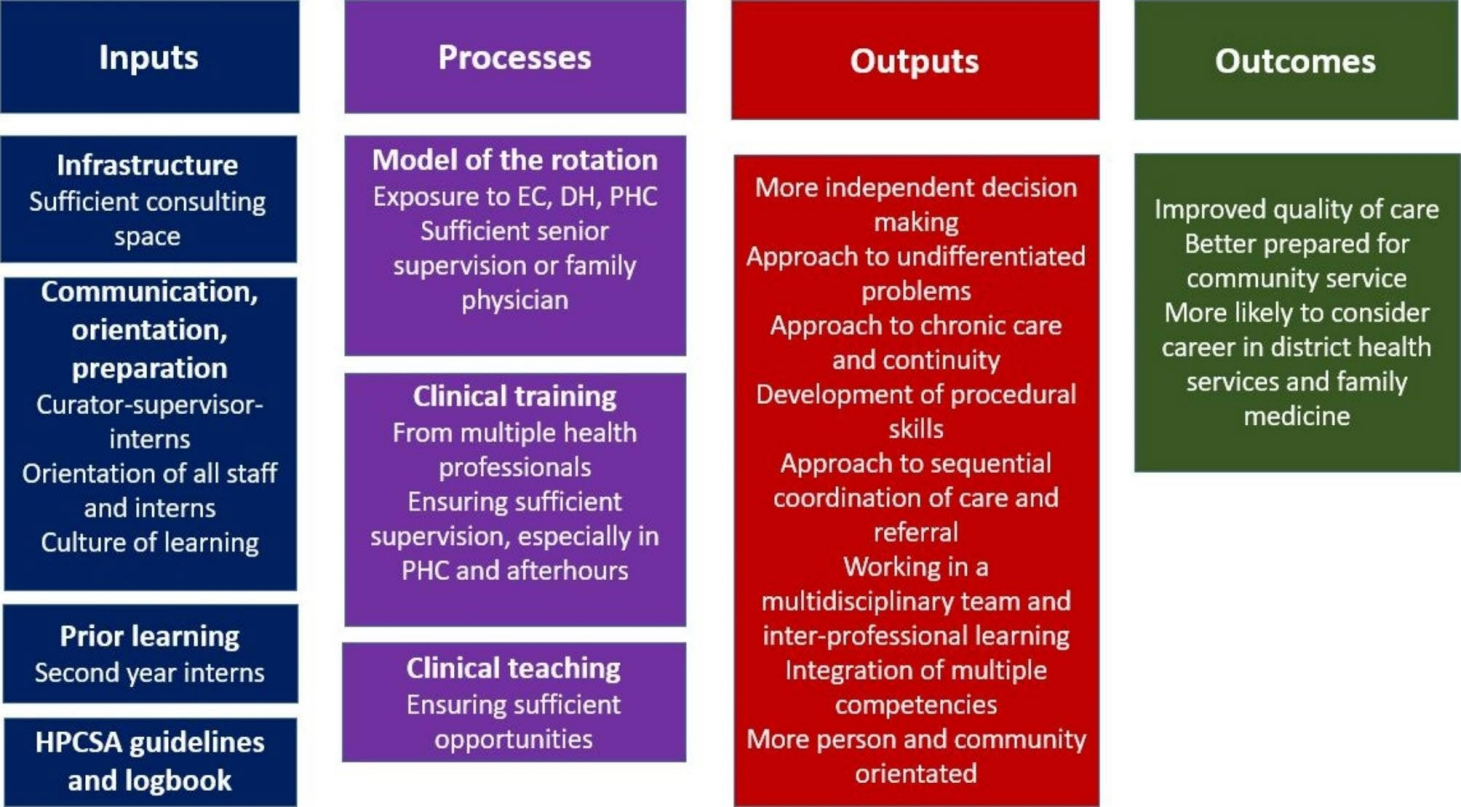
Summary of key findings as a programme theory [16]

The key findings can be organised into a programme theory as shown in Fig. 1 [16]. This identifies the key inputs and processes to make a success of the internship programme and the suggested outputs and outcomes.

Key inputs relate to the infrastructure, orientation of staff and interns, communication mechanisms between the curator-supervisors and interns, training of supervisors in basic skills for clinical training, the underlying learning environment, the guidance of the HPCSA logbook and value of prior learning in the first year of internship.

Key processes relate to the model of training in terms of how interns are allocated to PHC versus district hospital versus emergency centres in regional hospitals. It was clear that exposure to specialised services such as TB hospitals was not regarded positively. Exposure to PHC was favoured over the regional hospital EC. The model required the presence of senior supervisors and ideally a family physician who could coordinate, role model, share expertise, attract registrars with a training orientation, and develop a learning environment [17]. Clinical training should be from multiple health professionals, especially medical officers. Sufficient supervision in PHC and afterhours was an issue in some settings. Clinical teaching needed a dedicated programme or at least participation in the facility’s usual learning activities.

The respondents identified an important range of outputs that could be anticipated from these processes. These included more independent decision making, an approach to undifferentiated problems, chronic care and continuity, as well as development of procedural skills. Interns learnt about sequential coordination of care, making referrals, and working as part of a multidisciplinary team. They had to integrate knowledge and skills that were learnt in different disciplinary siloes and became more person and community-orientated. Overall, the respondents believed that these interns would be better prepared for community-service and more likely to consider a career within the district health services. The expectation would be that patient adverse incidents during the community-service year should diminish [13]. There was a suggestion that the addition of interns to clinical teams improved the quality of care overall through a reduction in workload for everyone, more time for patients and better cover afterhours. This was despite the need for support, supervision and some issues with professionalism.

### Discussion of key findings and implications

The new internship programme is a complex health intervention, and the Medical Research Council in the UK has suggested core elements to consider: the context, the programme theory, the stakeholders, key uncertainties, need to refine the intervention and economic considerations [16]. These topics will be used to further discuss the findings.

This study was undertaken in the *context* of COVID-19, which may have distorted the intended experience for many interns. COVID-19 led to the re-organization of many health services and reduced attention to training [18]. This was the first year that large numbers of interns were placed simultaneously on the district health platform and many role players were dealing with this for the first time. Change management was needed and all stakeholders were learning how to implement the rotation. For example, one district seemed to regard the interns more like students and refused to accept them. The Western Cape health system may also be a different context to other provinces. For example, the Western Cape has deployed many more family physicians in the district health services, which might improve the supervisory capacity [19].

*The programme theory* that emerges from the study (Fig. 1) can be compared with the espoused theory from the HPCSA [20]. Broadly speaking the rotation appears to deliver on the outcomes expected by the HPCSA: to manage undifferentiated conditions in primary care; provide chronic care; manage typical conditions in a district hospital; collaborate with other health professionals and integrate all medical knowledge. The overall impression is that the interns and supervisors preferred the longer rotation, as compared to the previous shorter 3-month rotation. The managers, supervisors and FPs were able to provide more insight, as they can compare experience with both.

*The stakeholders* were identified as the interns, their supervisors, the curators of the intern programmes and facility managers. Other stakeholders that emerged included medical officers and nurses. The HPCSA’s expectation is that supervision should be given by family physicians, specialists and medical officers with at least three years’ experience. While most respondents reported adequate and regular supervision by family physicians, there was still a concern about supervision in PHC and after hours in ECs. This seems to be an improvement from previous studies which reported that 31% of the time interns were supervised by a medical officer with less than three years’ experience [12]. A follow up study will quantify the issues found here.

*Key uncertainties* included the ideal allocation of time between settings, which was not clear from the findings, and all permutations appeared to work. There was a suggestion that there was too much exposure to the ECs in regional hospitals and that PHC exposure was more important. It was also not clear if more time should be spent on learning about clinical governance, improving the quality of care and patient safety, or other competencies other than direct patient care. Mentoring of these young doctors was not strong and it was not clear if this was an important deficiency. Interns are in a stressful transition period, working under considerable pressure and in a part of their careers and lifecycle where they may be embarking on marriage or more permanent partners. The long working hours, steep learning curve, high burden of disease, and lack of adequate resources all call for resilience and skills in dealing with uncertainty [21]. This would suggest that mentoring (and resilience training) for interns might be important. Due to the large distances between base and district hospitals, particularly in the rural districts, some interns had to move from the base hospital to another area. This had an impact on accommodation and personal costs, which the interns had to bear.

The findings suggest that future programs should *refine the intervention* by avoiding specialised allocations, reducing EC time, and ensuring more PHC time. There is a need to train the supervisors in workplace based educational skills. Family physicians are uniquely positioned to offer appropriate supervision, cost-effective role modelling and leadership in a multi-disciplinary team [22]. This could be an improvement from previous reports, where 25% of interns had an event that needed a senior doctor who was not available [8] It is important to select sites with adequate working space, the presence of a family physician, and with a culture of learning. This would go a long way to correct the experiences of inadequate facilities described previously. 12 Another priority is ensuring a well-structured clinical teaching programme. Other positive changes to improve the working and learning experiences of interns include adjusting the HPCSA logbook as the tool to guide their rotation; having clear roles for interns, community service doctors, registrars and how their different learning needs are enabled; exposure to clinical governance; and the contributions of inter-professional teams to district health services. Linking interns to outreaching specialists would build relationships between referral hospitals, improving patient referrals.

South Africa is in an *economic* crisis, with the gross domestic product growth outlook projected at 0.3% [23] With the Health Professions Training and Development Grant (HPTDG) being cut, the Department of Health may not be able to afford the number of interns and three years of compulsory posts for all graduates. Globally, countries like Israel are planning to reduce internship to six months in a bid to relieve doctor shortages [24]. Interns may however be cost effective for the district health services as they are paid for from the separate HPTDG. In the districts, their effect on improved quality of care, reduced workload and after hour cover, suggests a good return on investment.

### Strengths and limitations

There was acceptable inclusion of intended respondents in the Western Cape and saturation of data was reached. A limitation may be that information from community service doctors, junior medical officers and registrars regarding the experience of the interns is not known, as they were not included as participants in this study.

The lessons learnt and summarised in the programme theory (Fig. 1) are transferable to other provinces, understanding that the Western Cape health system may have more resources, resulting in a different context. The findings might not be as positive in other provinces and further research is required to evaluate this.

## Conclusions

The longer family medicine rotation was positively experienced by interns and supervisors. Exposure to district health services and PHC enabled interns to meet the outcomes expected by the HPCSA. A variety of structures for intern rotations in family medicine were found, appropriate to different contexts, with no clear optimal model. The interns were welcomed into health teams and added value to service delivery. There was better preparedness for community service. Supervision of interns was available at all sites, but differed in terms of intensity and quality. Family physicians were important as coordinators, supervisors, role models and custodians of the learning environment. There was a lack of mentorship services available to intern doctors and some confusion between roles of curators, supervisors and managers. Exposure to clinical governance was limited.

### Electronic supplementary material

Below is the link to the electronic supplementary material.

Supplementary Material 1

Supplementary Material 2

## Data Availability

Data is available in the Department of Family and Emergency Medicine, Stellenbosch University, contact number + 27 21 9,389,168, upon reasonable request.

## List of Abbreviations

PHC: Primary health care
ASSAF: Academy of Science of South Africa
HPCSA: Heath Professions Council of South Africa
EC: Emergency Centre
CPD: Continuing Professional Development
HPTDG: Health Professions Training and Development Grant

## Declarations

### Disclaimer

The views expressed in the submitted article are those of the authors and not an official position of an institution or funder.
