# Supplementary material for: Medical interns in district health services: an evaluation of the new family medicine rotation in the Western Cape of South Africa
Source: BMC Med Educ. 2023 Sep 4;23:636. doi: 10.1186/s12909-023-04605-6 (PMC10478251; doi:10.1186/s12909-023-04605-6)
Supplement: Supplementary file 1 — Supplementary Material 1 [file 12909_2023_4605_MOESM1_ESM.docx]

**Appendix A:**

**Interview guide for Interns:**

**Welcome and introduction:** Welcome to this interview. Today we will be exploring your experience of the 6 month family medicine rotation that you have completed as part of the 2^nd^ year of your internship. The interview will cover discussions around the model used at your site, learning that has taken place, supervision and how this rotation contributed to the health services in your district. The interview will be taped, but will be confidential.

**Opening statement:**

To begin, can you give your overall general impression of the new 6 month family medicine rotation that you have completed this year, including when and where was your rotation was held?

| **THEME** | **Important topics to explore:** | **Question guide for the interview:** |
| --- | --- | --- |
| Models | 1. Structure of the programme 2. Presence of orientation 3. Learning opportunities at the allocated site 4. Comparison to other rotations in internship 5. General flow/clinical allocations within the 6 months | 1. Describe the 6 month family medicine rotation in your 2^nd^ year of internship. 2. How do you feel your experience compared to colleagues at other sites; as well as to the rest of your internship? |
| Learning | 1. The contribution of the local environment to learning 2. HPCSA logbooks as guides to direct learning 3. Skills not achieved 4. Personal growth 5. Participation in reflection 6. Attitudes towards family medicine | 1. Describe your learning expectations for this rotation and how they changed over the 6 months. 2. Do you feel the learning that has taken place has adequately prepared you for community service? 3. Explain how the HPCSA logbook and outcomes contributed to your learning. 4. Are there ways in which the rotation can be improved, to enhance personal and clinical development as a doctor? |
| Supervision | 1. The role/relationship with the intern curators 2. The role/relationship with the intern supervisor 3. The presence of role models in the local teams 4. Available support: clinical and personal, during work hours and after hours 5. Short falls in supervision | 1. Can you describe the type and quality of supervision that you received during the 6 months. 2. Comment on mentorship and role models in the district health team. |
| Health services | 1. Impact on health services and delivery 2. Views of other staff members on the addition of interns 3. Balancing HPCSA expectations and clinical work | 1. Comment on your contribution to the health services, considering clinical and non-clinical experience gained. 2. Expand on any issues between HPCSA logbook expectations and the practicality of the job. |

**Appendix B:**

**Interview guide for intern curators/supervisors/managers:**

**Welcome and introduction:** Welcome to this interview. Today we will be exploring your experience of the new 6 month family medicine rotation that has recently been introduced at your facility. The interview will cover discussions around the model used at your site, learning that has taken place among interns, supervision and how this rotation contributed to the health services in your district. The interview will be taped, but will be confidential.

**Opening statement:**

To begin, can you give your overall general impression of the new 6 month family medicine rotation, including your role in the rotation at your facility?

| **THEME** | **Important topics:** | **Question guide for the interview:** |
| --- | --- | --- |
| Models | 1. Model design 2. Guidelines followed 3. Responsible persons (curators, managers, supervisors) 4. HPCSA expectations | 1. Describe the internship model at your location, and what influenced its setup and design. 2. How did your model align with the HPCSA requirements? |
| Learning | 1. HPCSA logbook – goals achieved / not achieved 2. Barriers to learning 3. Community service preparedness 4. Educational approach (formal tutorials, educational meetings, clinical training) 5. Non-clinical training 6. Impact on other students - registrars and students | 1. Describe the clinical and personal growth that was witnessed among the interns over the 6 months. 2. How easy was it to ensure exposure to necessary clinical and non-clinical skills, according to the logbook? 3. What types of learning and supervision took place at your site? |
| Supervision | 1. Preparedness of supervisors (formal training, orientation, guidance) 2. Contribution of family physicians to supervision within the team. 3. Availability of supervision and assistance 4. Strengths and weaknesses of supervision | 1. Comment on your involvement with the interns and your overall experience of this rotation, with regards to your role in supervision. 2. Describe the type and quality of supervision that was given to interns at your site. 3. Are there ways in which supervision can be improved for future groups? |
| Health services | 1. Impact on service delivery (patient care, effect on waiting times, clinical inexperience, professionalism, relationship with referral hospital, use of space) 2. Impressions from other staff members (allied, nursing etc) and patients, on the new addition of interns | 1. Comment on the impact of interns with regards to health services and district health teams, within your location. |
