# Supplementary material for: Medical interns in district health services: an evaluation of the new family medicine rotation in the Western Cape of South Africa
Source: BMC Med Educ. 2023 Sep 4;23:636. doi: 10.1186/s12909-023-04605-6 (PMC10478251; doi:10.1186/s12909-023-04605-6)

PARTICIPANT INFORMATION LEAFLET AND CONSENT FORM

| **TITLE OF RESEARCH PROJECT:** | |
| --- | --- |
| **Medical interns in district health services: an evaluation of the new family medicine rotation in the Western Cape, South Africa.** | |
| **DETAILS OF PRINCIPAL INVESTIGATOR (PI):** | |
| **Title, first name, surname:**  **Dr Lauren Hutton** | **Ethics reference number:**  **22709** |
| **Full postal address: Suite 2 Private Bag X013 Knysna 6571** | **PI Contact number:**  **0832369794** |

We would like to invite you to take part in a research project. Please take some time to read the information presented here, which will explain the details of this project. Please ask the study staff any questions about any part of this project that you do not fully understand. It is very important that you are completely satisfied that you clearly understand what this research entails and how you could be involved. Also, your participation is **entirely voluntary** and you are free to decline to participate. In other words, you may choose to take part, or you may choose not to take part. Nothing bad will come of it if you say no: it will not affect you negatively in any way whatsoever. Refusal to participate will involve no penalty or loss of benefits. You are also free to withdraw from the study at any point, even if you do agree to take part initially.

The Health Research Ethics Committee at Stellenbosch University has approved this study**.** The study will be conducted according to the ethical guidelines and principles of the international Declaration of Helsinki, the South African Guidelines for Good Clinical Practice (2006), the Medical Research Council (MRC) Ethical Guidelines for Research (2002), and the Department of Health Ethics in Health Research: Principles, Processes and Studies (2015).

**What is the purpose of the study?**

The South African Internship training for newly qualified medical doctors has undergone changes in 2020 and one of the most noticeable changes is the additional time in the Family Medicine domain. To this end a study to evaluate this change in rotation is taking place. The research will be divided into 2 phases, where the outcome of phase 1 will inform the research tool used in phase 2.

**Why have you been invited?**

In phase 1 the study will include a range of supervisors of interns and interns themselves. Through interviews key elements of intern training will be explored. The outcomes of the interviews will assist in creating a tool to evaluate the training across a broad selection of supervisors and interns in the Western Cape.

**Do I have to take part?**

No, you do not need to take part.

**What will happen if I take part?**

You will be asked to complete a consent form.

You will then be approached to participate in an interview with the researcher or research assistant, at a time that is convenient to you. The researcher or research assistant will not be from the same intern complex in which you work. This interview should last between 30 and 60 minutes. The interview could be done in person or using a secure online format, such as Zoom, if that is more convenient for you. The interview will be recorded and transcribed to be analyzed. The recordings will be held by the researchers and confidentiality will be maintained.

The responses in the interviews will be used to create a tool to evaluate the intern program across the Western Cape Province. There is a possibility that final results of this study will be combined to form part of similar research in other provinces, as part of a national study.

**Are there benefits to this study?**

With the change to the intern program in 2020, there is no research to evaluate the rotation. Your involvement will help to guide ongoing intern training.

**What are the risks of taking part?**

Confidentiality will be maintained. No part of your interview will be fed back to your hospital complex directly. The outcome of the study will be fed back, but the responses will be anonymized. Your participation and your responses will not affect your work or professional relationships in any way. There is minimal risk to you, in taking part.

**Will I be reimbursed for my participation?**

There is no reimbursement for this study.

**What will happen at the end of this study?**

At the end of Phase 1 the researchers will analyze the interviews and create a tool to evaluate the intern program. The results of Phase 1 will not be fed back to you, but the overall results of the study will be.

**Who is conducting this research study?**

This study is a collaboration between Stellenbosch University and the University of Cape Town, Departments of Family Medicine. Please see below for Principle investigators at each university.

**Is there anything else that you should know or do?**

- You can phone Dr Lauren Hutton at 0832369794 if you have any further queries or encounter any problems.
- You can phone the Health Research Ethics Committee at 021 938 9677/9819 if there still is something that your study doctor has not explained to you, or if you have a complaint.
- You will receive a copy of this information and consent form for you to keep safe.

### Declaration by participant

By signing below, I …………………………………..…………. agree to take part in a research study entitled **Medical interns in district health services: an evaluation of the new family medicine rotation in the Western Cape, South Africa.**.

I declare that:

- I have read this information and consent form, or it was read to me, and it is written in a language in which I am fluent and with which I am comfortable.
- I have had a chance to ask questions and I am satisfied that all my questions have been answered.
- I understand that taking part in this study is **voluntary,** and I have not been pressured to take part.
- I may choose to leave the study at any time and nothing bad will come of it – I will not be penalised or prejudiced in any way.
- I may be asked to leave the study before it has finished, if the researcher feels it is in my best interests, or if I do not follow the study plan that we have agreed on.

Signed at (*place*) ......................…........…………….. on (*date*) …………....……….. 2021.

**Signature of participant Signature of witness**

### Declaration by investigator

I *(name)* ……………………………………………..……… declare that:

- I explained the information in this document in a simple and clear manner to …………………………………..
- I encouraged him/her to ask questions and took enough time to answer them.
- I am satisfied that he/she completely understands all aspects of the research, as discussed above.
- I did/did not use an interpreter. (*If an interpreter is used then the interpreter must sign the declaration below.)*

Signed at (*place*) ......................…........…………….. on (*date*) …………....……….. 2021.

**Signature of investigator Signature of witness**

**Permission to have all anonymous data shared with journals:**

*Please carefully read the statements below (or have them read to you) and think about your choice. No matter what you decide, it will not affect whether you can be in the research study, or your routine health care*

When this study is finished, we would like to publish results of the study in journals. Most journals require us to share your anonymous data with them before they publish the results. Therefore, we would like to obtain your permission to have your anonymous data shared with journals.

**Tick the Option you choose for anonymous data sharing with journals:**

I agree to have my anonymous data shared with journals during publication of results of this study

Signature____________



OR

I do not agree to have my anonymous data shared with journals during publication of results of this study

Signature____________


Supplement: Supplementary file 2 — Supplementary Material 2 [file 12909_2023_4605_MOESM2_ESM.docx]
